# The 10th year of the *Journal of Cachexia, Sarcopenia and Muscle*

**DOI:** 10.1002/jcsm.12657

**Published:** 2020-12-19

**Authors:** Markus S. Anker, Jochen Springer, Andrew JS Coats, Stephan von Haehling

**Affiliations:** ^1^ Division of Cardiology and Metabolism, Department of Cardiology (CVK) Charité University Medicine Berlin Berlin Germany; ^2^ Berlin Institute of Health Center for Regenerative Therapies (BCRT) Berlin Germany; ^3^ DZHK (German Centre for Cardiovascular Research), partner site Berlin Berlin Germany; ^4^ Department of Cardiology, Campus Benjamin Franklin (CBF) Charité University Medicine Berlin Berlin Germany; ^5^ Department of Cardiology IRCCS San Raffaele Pisana Rome Italy; ^6^ Department of Cardiology and Pneumology University Medical Center Göttingen Göttingen Germany; ^7^ DZHK (German Centre for Cardiovascular Research), partner site Göttingen Göttingen Germany

The *Journal of Cachexia, Sarcopenia and Muscle* (JCSM) is an open access peer‐reviewed journal that was founded 10 years ago in 2010. It is published by Wiley and in association with the non‐profit organization ‘Society on Sarcopenia, Cachexia, and Wasting Disorders (SCWD^1^)’. Currently, the journal is published six times per year. JCSM attracts research from many different countries such as the USA, Germany, England, Italy, Netherlands, Australia, France, Canada, China, and Switzerland (top 10 countries where submissions originated from in 2019). Editor‐in‐chief is Prof. Stefan Anker (Berlin, Germany), Co‐Editor‐in‐Chief is Prof. Stephan von Haehling (Göttingen, Germany), and Senior Consulting Editor is Prof. Andrew Coats (Rome, Italy). The editorial office is led by Monika Diek and Corinna Denecke, without whom this journal could not be published in time. This year JCSM has received 642 submissions so far (until October 2020). Certainly, it is only possible to evaluate such a large amount of submissions together with a great team of associate editors and reviewers. All these researchers work closely together with the goal to publish excellent new original papers, reviews, and editorials in the field of wasting diseases.

JCSM's main areas of interest include the loss of body weight with regard to cachexia, malnourishment, anorexia, and lipolysis, as well as loss of muscle mass in sarcopenia and muscle wasting. Most original articles focus on preclinical investigations with regard to a better mechanistic understanding of the underlying pathophysiology; clinical investigations and biomarker assessment in patients with different wasting diseases; and epidemiological questions regarding the general burden of wasting diseases and its impact on other co‐morbidities. Since cachexia and sarcopenia often occur in the setting of other chronic diseases, many different illnesses are thematized in the journal, including cancer, heart failure, and other cardiovascular diseases, chronic kidney disease, chronic obstructive pulmonary disease, neurological diseases, and many others. There is also great interest in clinical studies, presenting the results of randomized, placebo controlled trials. Another field of interest includes age‐related changes of body composition and its impact on patients' quality of life, endurance, morbidity, and mortality. JCSM targets the entire scientific community including biologists, pharmacologists, physicists, biochemists, physicians, clinicians, trialists, dieticians, nurses, students, and basic scientists.

In 2019, Web of Science ranked JCSM number 2/51 in the section ‘Geriatrics & Gerontology’ and number 9/165 in the section ‘Medicine, General & Internal’ in 2019.^2^ One of the most important measures for any research journal is the Thomson Scientific journal impact. The 2019 Thomson Scientific journal impact factor for JCSM was calculated by counting all citations in 2019 to papers published in JCSM in 2017 (987 citations) and 2018 (699 citations) and dividing it by the number of citable items published in JCSM in 2017 (*n* = 82) and 2018 (*n* = 90). Hence, the 2019 Thomson Scientific journal impact factor for JCSM was 9.802 (1686/172). The number of total citations towards JCSM is increasing each year [2013: 516 citations, 2014: 713 citations (38%), 2015: 901 citations (26%), 2016: 1310 (+45%), 2017: 2207 citations (+68%), 2018: 2799 citations (+27%), 2019: 3553 citations (+27%)].^2^ Some papers in the past have been heavily cited: the most cited paper ever published in JCSM was an editorial by von Haehling *et al*.^3^ published in 2010 about ‘facts and numbers’ with regard to cachexia. So far this paper has already been cited 521 times. With regard to the top 10 cited papers ever published in JCSM (*Table* 1), these publications on average received 189 citations (as of September 2020), and they were all published between 2010 and 2016. Interestingly, these top 10 cited papers included a variation of different publications with four reviews, three editorials, and three original articles. Such diversity is also found when looking at the top 20 cited papers published in 2017–19 (*Tables* 2, 3, 4) with 11 reviews, 5 editorials, and 44 original articles—even though most citations are towards original articles. The three best cited papers from each year (2017–19; nine papers) mainly addressed the following research areas: cachexia and malnourishment (three papers); sarcopenia, muscle function and mass (seven papers); and adipose tissue (one paper). This demonstrates that nowadays not only research into the field of cachexia and malnourishment is of great interest to the scientific community but also research into sarcopenia, muscle function and mass.

**Table 1:** JCSM top 10 cited articles of all time

Rank	First author	Title	Article type	Times cited
1	von Haehling, S^3^	Cachexia as a major underestimated and unmet medical need: facts and numbers	Editorial Material	521
2	Morley, JE^4^	Prevalence, incidence, and clinical impact of sarcopenia: facts, numbers, and epidemiology—update 2014	Editorial Material	192
3	Dalton, JT^5^	The selective androgen receptor modulator GTx‐024 (enobosarm) improves lean body mass and physical function in healthy elderly men and postmenopausal women: results of a double‐blind, placebo‐controlled phase II trial	Article	184
4	Malmstrom, TK^6^	SARC‐F: a symptom score to predict persons with sarcopenia at risk for poor functional outcomes	Article	166
5	Fanzani, A^7^	Molecular and cellular mechanisms of skeletal muscle atrophy: an update	Review	145
6	Cesari, M^8^	Biomarkers of sarcopenia in clinical trials—recommendations from the International Working Group on Sarcopenia	Article	140
7	Wakabayashi, H^9^	Rehabilitation nutrition for sarcopenia with disability: a combination of both rehabilitation and nutrition care management	Review	138
8	Lenk, K^10^	Skeletal muscle wasting in cachexia and sarcopenia: molecular pathophysiology and impact of exercise training	Review	136
9	Anker, SD^11^	Welcome to the ICD‐10 code for sarcopenia	Editorial Material	135
10	Elkina, Y^12^	The role of myostatin in muscle wasting: an overview	Review	134

**Table 2:** Top 20 cited articles published in 2019

Rank	First author	Title	Article type	Times cited
1	Cederholm, T^13^	GLIM criteria for the diagnosis of malnutrition—a consensus report from the global clinical nutrition community	Article	36
2	Bauer, J^14^	Sarcopenia: a time for action. An SCWD position paper	Article	35
3	Yeung, SSY^15^	Sarcopenia and its association with falls and fractures in older adults: a systematic review and meta‐analysis	Review	25
4	Pin, F^16^	Cachexia induced by cancer and chemotherapy yield distinct perturbations to energy metabolism	Article	21
5	Dolan, RD^17^	The relationship between computed tomography‐derived body composition, systemic inflammatory response, and survival in patients undergoing surgery for colorectal cancer	Article	20
6	Evans, WJ^18^	D_3_‐Creatine dilution and the importance of accuracy in the assessment of skeletal muscle mass	Review	19
7	Li, ZH^19^	LncIRS1 controls muscle atrophy via sponging miR‐15 family to activate IGF1‐PI3K/AKT pathway	Article	17
8	Okugawa, Y^20^	Circulating miR‐203 derived from metastatic tissues promotes myopenia in colorectal cancer patients	Article	14
9	Qazi, TH^21^	Cell therapy to improve regeneration of skeletal muscle injuries	Review	14
10	Ramirez‐Velez, R^22^	Reference values for handgrip strength and their association with intrinsic capacity domains among older adults	Article	13
11	Dijksterhuis, WPM^23^	Association between body composition, survival, and toxicity in advanced esophagogastric cancer patients receiving palliative chemotherapy	Article	13
12	Rodriguez‐Manas, L^24^	Effectiveness of a multimodal intervention in functionally impaired older people with type 2 diabetes mellitus	Article	12
13	Oost, LJ^25^	Fibroblast growth factor 21 controls mitophagy and muscle mass	Article	12
14	ten Haaf, DSM^26^	Protein supplementation improves lean body mass in physically active older adults: a randomized placebo‐controlled trial	Article	12
15	Naito, T^27^	Feasibility of early multimodal interventions for elderly patients with advanced pancreatic and non‐small‐cell lung cancer	Article	12
16	Lee, J^28^	Muscle radiodensity loss during cancer therapy is predictive for poor survival in advanced endometrial cancer	Article	10
17	Hong, Y^29^	Amelioration of muscle wasting by glucagon‐like peptide‐1 receptor agonist in muscle atrophy	Article	9
18	Hughes, MC^30^	Early myopathy in Duchenne muscular dystrophy is associated with elevated mitochondrial H_2_O_2_ emission during impaired oxidative phosphorylation	Article	9
19	Zhu, XX^31^	MyD88 signalling is critical in the development of pancreatic cancer cachexia	Article	9
20	Koppe, L^32^	Kidney cachexia or protein‐energy wasting in chronic kidney disease: facts and numbers	Editorial Material	8

**Table 3:** Top 20 cited articles published in 2018

Rank	First author	Title	Article type	Times cited
1	Buckinx, F^33^	Pitfalls in the measurement of muscle mass: a need for a reference standard	Article	113
2	Tieland, M^34^	Skeletal muscle performance and ageing	Review	95
3	Daly, LE^35^	Loss of skeletal muscle during systemic chemotherapy is prognostic of poor survival in patients with foregut cancer	Article	45
4	Zhang, ZK^36^	A newly identified lncRNA MAR1 acts as a miR‐487b sponge to promote skeletal muscle differentiation and regeneration	Article	34
5	Mucke, M^37^	Systematic review and meta‐analysis of cannabinoids in palliative medicine	Review	32
6	Choi, MH^38^	Sarcopenia is negatively associated with long‐term outcomes in locally advanced rectal cancer	Article	29
7	Mayr, R^39^	Sarcopenia as a comorbidity‐independent predictor of survival following radical cystectomy for bladder cancer	Article	28
8	Zhang, AQ^40^	miRNA‐23a/27a attenuates muscle atrophy and renal fibrosis through muscle‐kidney crosstalk	Article	26
9	Ebadi, M^41^	Poor performance of psoas muscle index for identification of patients with higher waitlist mortality risk in cirrhosis	Article	25
10	Choi, MH^42^	Preoperative sarcopenia and post‐operative accelerated muscle loss negatively impact survival after resection of pancreatic cancer	Article	25
11	Rhee, CM^43^	Low‐protein diet for conservative management of chronic kidney disease: a systematic review and meta‐analysis of controlled trials	Review	25
12	Brown, JC^44^	The evolution of body composition in oncology‐epidemiology, clinical trials, and the future of patient care: facts and numbers	Editorial Material	24
13	Brzeszczynska, J^45^	Alterations in the in vitro and in vivo regulation of muscle regeneration in healthy ageing and the influence of sarcopenia	Article	22
14	Siracusa, J^46^	Circulating myomiRs: a new class of biomarkers to monitor skeletal muscle in physiology and medicine	Review	22
15	Nissinen, TA^47^	Treating cachexia using soluble ACVR2B improves survival, alters mTOR localization, and attenuates liver and spleen responses	Article	21
16	Connolly, M^48^	miR‐424‐5p reduces ribosomal RNA and protein synthesis in muscle wasting	Article	21
17	Grimm, A^49^	Repeatability of Dixon magnetic resonance imaging and magnetic resonance spectroscopy for quantitative muscle fat assessments in the thigh	Article	20
18	Brown, JC^50^	The deterioration of muscle mass and radiodensity is prognostic of poor survival in stage I–III colorectal cancer: a population‐based cohort study (C‐SCANS)	Article	20
19	Kays, JK^51^	Three cachexia phenotypes and the impact of fat‐only loss on survival in FOLFIRINOX therapy for pancreatic cancer	Article	20
20	Xiao, JJ^52^	Associations of pre‐existing co‐morbidities with skeletal muscle mass and radiodensity in patients with non‐metastatic colorectal cancer	Article	20

**Table 4:** Top 20 cited articles published in 2017

Rank	First author	Title	Article type	Times cited
1	Kalafateli, M^53^	Malnutrition and sarcopenia predict post‐liver transplantation outcomes independently of the Model for End‐stage Liver Disease score	Article	84
2	Solheim, TS^54^	A randomized phase II feasibility trial of a multimodal intervention for the management of cachexia in lung and pancreatic cancer	Article	74
3	Boengler, K^55^	Mitochondria and ageing: role in heart, skeletal muscle and adipose tissue	Review	69
4	van Dijk, DPJ^56^	Low skeletal muscle radiation attenuation and visceral adiposity are associated with overall survival and surgical site infections in patients with pancreatic cancer	Article	64
5	Nijholt, W^57^	The reliability and validity of ultrasound to quantify muscles in older adults: a systematic review	Review	62
6	van Vugt, JLA^58^	A comparative study of software programmes for cross‐sectional skeletal muscle and adipose tissue measurements on abdominal computed tomography scans of rectal cancer patients	Article	62
7	Rutten, IJG^59^	Psoas muscle area is not representative of total skeletal muscle area in the assessment of sarcopenia in ovarian cancer	Article	59
8	Martone, AM^60^	The incidence of sarcopenia among hospitalized older patients: results from the Glisten study	Article	58
9	Holecek, M^61^	Beta‐hydroxy‐beta‐methylbutyrate supplementation and skeletal muscle in healthy and muscle‐wasting conditions	Review	57
10	Snijders, T^62^	Muscle fibre capillarization is a critical factor in muscle fibre hypertrophy during resistance exercise training in older men	Article	52
11	Nishikawa, H^63^	Elevated serum myostatin level is associated with worse survival in patients with liver cirrhosis	Article	47
12	Brown, JL^64^	Mitochondrial degeneration precedes the development of muscle atrophy in progression of cancer cachexia in tumour‐bearing mice	Article	46
13	Morley, JE^65^	Anorexia of ageing: a key component in the pathogenesis of both sarcopenia and cachexia	Editorial Material	45
14	Bye, A^66^	Muscle mass and association to quality of life in non‐small cell lung cancer patients	Article	43
15	St‐Jean‐Pelletier, F^67^	The impact of ageing, physical activity, and pre‐frailty on skeletal muscle phenotype, mitochondrial content, and intramyocellular lipids in men	Article	43
16	Baracos, VE^68^	Psoas as a sentinel muscle for sarcopenia: a flawed premise	Editorial Material	42
17	Gonzalez, MC^69^	Bioelectrical impedance analysis for diagnosing sarcopenia and cachexia: what are we really estimating?	Editorial Material	42
18	Mochamat Cuhls, H^70^	A systematic review on the role of vitamins, minerals, proteins, and other supplements for the treatment of cachexia in cancer: a European Palliative Care Research Centre cachexia project	Review	40
19	Tournadre, A^71^	Changes in body composition and metabolic profile during interleukin 6 inhibition in rheumatoid arthritis	Article	39
20	dos Santos, L^72^	Sarcopenia and physical independence in older adults: the independent and synergic role of muscle mass and muscle function	Article	38

## Conflict of interest

None declared.

## References

[1] Cachexia ‐ Society on Sarcopenia, Cachexia and Wasting Disorders. https://society‐scwd.org/. Accessed October 1, 2020.

[2] Web of Knowledge. http://www.webofknowledge.com/. Accessed October 1, 2020.

[3] von Haehling S , Anker SD . Cachexia as a major underestimated and unmet medical need: facts and numbers. September 2010. 2010;1:1–5. 10.1007/s13539-010-0002-6 PMC3060651 21475699

[4] Morley JE , Anker SD , von Haehling S . Prevalence, incidence, and clinical impact of sarcopenia: facts, numbers, and epidemiology—update 2014. December 2014. 2014;5:253–259. 10.1007/s13539-014-0161-y PMC4248415 25425503

[5] Dalton JT , Barnette KG , Bohl CE , Hancock ML , Rodriguez D , Dodson ST , et al. The selective androgen receptor modulator GTx‐024 (enobosarm) improves lean body mass and physical function in healthy elderly men and postmenopausal women: results of a double‐blind, placebo‐controlled phase II trial. J Cachexia Sarcopenia Muscle, 2011;2:153–161. 22031847 10.1007/s13539-011-0034-6 PMC3177038

[6] Malmstrom TK , Miller DK , Simonsick EM , Ferrucci L , Morley JE . SARC‐F: a symptom score to predict persons with sarcopenia at risk for poor functional outcomes. J Cachexia Sarcopenia Muscle 2016;7:28–36. 27066316 10.1002/jcsm.12048 PMC4799853

[7] Fanzani A , Conraads VM , Penna F , Martinet W . Molecular and cellular mechanisms of skeletal muscle atrophy: an update. J Cachexia Sarcopenia Muscle, September 2012 2012;3:163–179. 22673968 10.1007/s13539-012-0074-6 PMC3424188

[8] Cesari M , Fielding RA , Pahor M , Goodpaster B , Hellerstein M , Kan V , et al. Biomarkers of sarcopenia in clinical trials—recommendations from the International Working Group on Sarcopenia. J Cachexia Sarcopenia Muscle. September 2012 2012;3:181–190. 22865205 10.1007/s13539-012-0078-2 PMC3424187

[9] Wakabayashi H , Sakuma K . Rehabilitation nutrition for sarcopenia with disability: a combination of both rehabilitation and nutrition care management. J Cachexia Sarcopenia Muscle, December 2014 2014;5:269–277. 25223471 10.1007/s13539-014-0162-x PMC4248414

[10] Lenk K , Schuler G , Adams V . Skeletal muscle wasting in cachexia and sarcopenia: molecular pathophysiology and impact of exercise training. September 2010. J Cachexia Sarcopenia Muscle 2010;1:9–21. 21475693 10.1007/s13539-010-0007-1 PMC3060644

[11] Anker SD , Morley JE , von Haehling S . Welcome to the ICD‐10 code for sarcopenia. J Cachexia Sarcopenia Muscle 2016;7:512–514. 27891296 10.1002/jcsm.12147 PMC5114626

[12] Elkina Y , von Haehling S , Anker SD , Springer J . The role of myostatin in muscle wasting: an overview. J Cachexia Sarcopenia Muscle. September 2011 2011;2:143–151. 21966641 10.1007/s13539-011-0035-5 PMC3177043

[13] Cederholm T , Jensen GL , Correia MITD , Gonzalez MC , Fukushima R , Higashiguchi T , et al. GLIM criteria for the diagnosis of malnutrition—a consensus report from the global clinical nutrition community. J Cachexia Sarcopenia Muscle 2019;10:207–217. 30920778 10.1002/jcsm.12383 PMC6438340

[14] Bauer J , Morley JE , Schols AMWJ , Ferrucci L , Cruz‐Jentoft AJ , Dent E , et al. Sarcopenia: a time for action. An SCWD position paper. J Cachexia Sarcopenia Muscle 2019;10:956–961. 31523937 10.1002/jcsm.12483 PMC6818450

[15] Yeung SSY , Reijnierse EM , Pham VK , Trappenburg MC , Lim WK , Meskers CGM , et al. Sarcopenia and its association with falls and fractures in older adults: a systematic review and meta‐analysis. J Cachexia Sarcopenia Muscle 2019;10:485–500. 30993881 10.1002/jcsm.12411 PMC6596401

[16] Pin F , Barreto R , Couch ME , Bonetto A , O'Connell TM . Cachexia induced by cancer and chemotherapy yield distinct perturbations to energy metabolism. J Cachexia Sarcopenia Muscle 2019;10:140–154. 30680954 10.1002/jcsm.12360 PMC6438345

[17] Dolan RD , Almasaudi AS , Dieu LB , Horgan PG , McSorley ST , McMillan DC . The relationship between computed tomography‐derived body composition, systemic inflammatory response, and survival in patients undergoing surgery for colorectal cancer. J Cachexia Sarcopenia Muscle 2019;10:111–122. 30460764 10.1002/jcsm.12357 PMC6438413

[18] Evans WJ , Hellerstein M , Orwoll E , Cummings S , Cawthon PM . D_3_‐Creatine dilution and the importance of accuracy in the assessment of skeletal muscle mass. J Cachexia Sarcopenia Muscle 2019;10:14–21. 30900400 10.1002/jcsm.12390 PMC6438329

[19] Li Z , Cai B , Abdalla BA , Zhu X , Zheng M , Han P , et al. LncIRS1 controls muscle atrophy via sponging miR‐15 family to activate IGF1‐PI3K/AKT pathway. J Cachexia Sarcopenia Muscle 2019;10:391–410. 30701698 10.1002/jcsm.12374 PMC6463472

[20] Okugawa Y , Toiyama Y , Hur K , Yamamoto A , Yin C , Ide S , et al. Circulating miR‐203 derived from metastatic tissues promotes myopenia in colorectal cancer patients. J Cachexia Sarcopenia Muscle 2019;10:536–548. 31091026 10.1002/jcsm.12403 PMC6596405

[21] Qazi TH , Duda GN , Ort MJ , Perka C , Geissler S , Winkler T . Cell therapy to improve regeneration of skeletal muscle injuries. J Cachexia Sarcopenia Muscle 2019;10:501–516. 30843380 10.1002/jcsm.12416 PMC6596399

[22] Ramírez‐Vélez R , Correa‐Bautista JE , García‐Hermoso A , Cano CA , Izquierdo M . Reference values for handgrip strength and their association with intrinsic capacity domains among older adults. J Cachexia Sarcopenia Muscle 2019;10:278–286. 30843369 10.1002/jcsm.12373 PMC6463468

[23] Dijksterhuis WPM , Pruijt MJ , van der Woude SO , Klaassen R , Kurk SA , van Oijen MGH , et al. Association between body composition, survival, and toxicity in advanced esophagogastric cancer patients receiving palliative chemotherapy. J Cachexia Sarcopenia Muscle 2019;10:199–206. 30666831 10.1002/jcsm.12371 PMC6438339

[24] Rodriguez‐Mañas L , Laosa O , Vellas B , Paolisso G , Topinkova E , Oliva‐Moreno J , et al. Effectiveness of a multimodal intervention in functionally impaired older people with type 2 diabetes mellitus. J Cachexia Sarcopenia Muscle 2019;10:721–733. 31016897 10.1002/jcsm.12432 PMC6711410

[25] Oost LJ , Kustermann M , Armani A , Blaauw B , Romanello V . Fibroblast growth factor 21 controls mitophagy and muscle mass. J Cachexia Sarcopenia Muscle 2019;10:630–642. 30895728 10.1002/jcsm.12409 PMC6596457

[26] ten Haaf DSM , Eijsvogels TMH , Bongers CCWG , Horstman AMH , Timmers S , de Groot LCPGM , et al. Protein supplementation improves lean body mass in physically active older adults: a randomized placebo‐controlled trial. J Cachexia Sarcopenia Muscle 2019;10:298–310. 30848096 10.1002/jcsm.12394 PMC6463466

[27] Naito T , Mitsunaga S , Miura S , Tatematsu N , Inano T , Mouri T , et al. Feasibility of early multimodal interventions for elderly patients with advanced pancreatic and non‐small‐cell lung cancer. J Cachexia Sarcopenia Muscle 2019;10:73–83. 30334618 10.1002/jcsm.12351 PMC6438328

[28] Lee J , Lin J‐B , Wu M‐H , Jan Y‐T , Chang C‐L , Huang C‐Y , et al. Muscle radiodensity loss during cancer therapy is predictive for poor survival in advanced endometrial cancer. J Cachexia Sarcopenia Muscle 2019;10:814–826. 31094101 10.1002/jcsm.12440 PMC6711455

[29] Hong Y , Lee JH , Jeong KW , Choi CS , Jun H‐S . Amelioration of muscle wasting by glucagon‐like peptide‐1 receptor agonist in muscle atrophy. J Cachexia Sarcopenia Muscle 2019;10:903–918. 31020810 10.1002/jcsm.12434 PMC6711418

[30] Hughes MC , Ramos SV , Turnbull PC , Rebalka IA , Cao A , Monaco CMF , et al. Early myopathy in Duchenne muscular dystrophy is associated with elevated mitochondrial H_2_O_2_ emission during impaired oxidative phosphorylation. J Cachexia Sarcopenia Muscle 2019;10:643–661. 30938481 10.1002/jcsm.12405 PMC6596403

[31] Zhu X , Burfeind KG , Michaelis KA , Braun TP , Olson B , Pelz KR , et al. MyD88 signalling is critical in the development of pancreatic cancer cachexia. J Cachexia Sarcopenia Muscle 2019;10:378–390. 30666818 10.1002/jcsm.12377 PMC6463469

[32] Koppe L , Fouque D , Kalantar‐Zadeh K . Kidney cachexia or protein‐energy wasting in chronic kidney disease: facts and numbers. J Cachexia Sarcopenia Muscle 2019 Jun;10:479–484. 30977979 10.1002/jcsm.12421 PMC6596400

[33] Buckinx F , Landi F , Cesari M , Fielding RA , Visser M , Engelke K , et al. Pitfalls in the measurement of muscle mass: a need for a reference standard. J Cachexia Sarcopenia Muscle 2018;9:269–278. 29349935 10.1002/jcsm.12268 PMC5879987

[34] Tieland M , Trouwborst I , Clark BC . Skeletal muscle performance and ageing. J Cachexia Sarcopenia Muscle 2018;9:3–19. 29151281 10.1002/jcsm.12238 PMC5803609

[35] Daly LE , Ní Bhuachalla ÉB , Power DG , Cushen SJ , James K , Ryan AM . Loss of skeletal muscle during systemic chemotherapy is prognostic of poor survival in patients with foregut cancer. J Cachexia Sarcopenia Muscle 2018;9:315–325. 29318756 10.1002/jcsm.12267 PMC5879982

[36] Zhang Z‐K , Li J , Guan D , Liang C , Zhuo Z , Liu J , et al. A newly identified lncRNA MAR1 acts as a miR‐487b sponge to promote skeletal muscle differentiation and regeneration. J Cachexia Sarcopenia Muscle 2018;9:613–626. 29512357 10.1002/jcsm.12281 PMC5989759

[37] Mücke M , Weier M , Carter C , Copeland J , Degenhardt L , Cuhls H , et al. Systematic review and meta‐analysis of cannabinoids in palliative medicine. J Cachexia Sarcopenia Muscle 2018;9:220–234. 29400010 10.1002/jcsm.12273 PMC5879974

[38] Choi MH , Oh SN , Lee IK , Oh ST , Won DD . Sarcopenia is negatively associated with long‐term outcomes in locally advanced rectal cancer. J Cachexia Sarcopenia Muscle 2018;9:53–59. 28849630 10.1002/jcsm.12234 PMC5803619

[39] Mayr R , Gierth M , Zeman F , Reiffen M , Seeger P , Wezel F , et al. Sarcopenia as a comorbidity‐independent predictor of survival following radical cystectomy for bladder cancer. J Cachexia Sarcopenia Muscle 2018;9:505–513. 29479839 10.1002/jcsm.12279 PMC5989852

[40] Zhang A , Li M , Wang B , Klein JD , Price SR , Wang XH . miRNA‐23a/27a attenuates muscle atrophy and renal fibrosis through muscle‐kidney crosstalk. J Cachexia Sarcopenia Muscle 2018;9:755–770. 29582582 10.1002/jcsm.12296 PMC6104113

[41] Ebadi M , Wang CW , Lai JC , Dasarathy S , Kappus MR , Dunn MA , et al. Poor performance of psoas muscle index for identification of patients with higher waitlist mortality risk in cirrhosis. J Cachexia Sarcopenia Muscle 2018;9:1053–1062. 30269421 10.1002/jcsm.12349 PMC6240754

[42] Choi MH , Yoon SB , Lee K , Song M , Lee IS , Lee MA , et al. Preoperative sarcopenia and post‐operative accelerated muscle loss negatively impact survival after resection of pancreatic cancer. J Cachexia Sarcopenia Muscle 2018;9:326–334. 29399990 10.1002/jcsm.12274 PMC5879976

[43] Rhee CM , Ahmadi S‐F , Kovesdy CP , Kalantar‐Zadeh K . Low‐protein diet for conservative management of chronic kidney disease: a systematic review and meta‐analysis of controlled trials. J Cachexia Sarcopenia Muscle 2018;9:235–245. 29094800 10.1002/jcsm.12264 PMC5879959

[44] Brown JC , Cespedes Feliciano EM , Caan BJ . The evolution of body composition in oncology—epidemiology, clinical trials, and the future of patient care: facts and numbers. J Cachexia Sarcopenia Muscle 2018;9:1200–1208. 30637983 10.1002/jcsm.12379 PMC6351674

[45] Brzeszczyńska J , Meyer A , McGregor R , Schilb A , Degen S , Tadini V , et al. Alterations in the in vitro and in vivo regulation of muscle regeneration in healthy ageing and the influence of sarcopenia. J Cachexia Sarcopenia Muscle 2018;9:93–105. 29214748 10.1002/jcsm.12252 PMC5803613

[46] Siracusa J , Koulmann N , Banzet S . Circulating myomiRs: a new class of biomarkers to monitor skeletal muscle in physiology and medicine. J Cachexia Sarcopenia Muscle 2018;9:20–27. 29193905 10.1002/jcsm.12227 PMC5803618

[47] Nissinen TA , Hentilä J , Penna F , Lampinen A , Lautaoja JH , Fachada V , et al. Treating cachexia using soluble ACVR2B improves survival, alters mTOR localization, and attenuates liver and spleen responses. J Cachexia Sarcopenia Muscle 2018;9:514–529. 29722201 10.1002/jcsm.12310 PMC5989872

[48] Connolly M , Paul R , Farre‐Garros R , Natanek SA , Bloch S , Lee J , et al. miR‐424‐5p reduces ribosomal RNA and protein synthesis in muscle wasting. J Cachexia Sarcopenia Muscle 2018;9:400–416. 29215200 10.1002/jcsm.12266 PMC5879973

[49] Grimm A , Meyer H , Nickel MD , Nittka M , Raithel E , Chaudry O , et al. Repeatability of Dixon magnetic resonance imaging and magnetic resonance spectroscopy for quantitative muscle fat assessments in the thigh. J Cachexia Sarcopenia Muscle 2018;9:1093–1100. 30221479 10.1002/jcsm.12343 PMC6240750

[50] Brown JC , Caan BJ , Meyerhardt JA , Weltzien E , Xiao J , Cespedes Feliciano EM , et al. The deterioration of muscle mass and radiodensity is prognostic of poor survival in stage I–III colorectal cancer: a population‐based cohort study (C‐SCANS). J Cachexia Sarcopenia Muscle 2018;9:664–672. 29766660 10.1002/jcsm.12305 PMC6104108

[51] Kays JK , Shahda S , Stanley M , Bell TM , O'Neill BH , Kohli MD , et al. Three cachexia phenotypes and the impact of fat‐only loss on survival in FOLFIRINOX therapy for pancreatic cancer. J Cachexia Sarcopenia Muscle 2018;9:673–684. 29978562 10.1002/jcsm.12307 PMC6104116

[52] Xiao J , Caan BJ , Weltzien E , Cespedes Feliciano EM , Kroenke CH , Meyerhardt JA , et al. Associations of pre‐existing co‐morbidities with skeletal muscle mass and radiodensity in patients with non‐metastatic colorectal cancer. J Cachexia Sarcopenia Muscle 2018;9:654–663. 29675984 10.1002/jcsm.12301 PMC6104112

[53] Kalafateli M , Mantzoukis K , Choi Yau Y , Mohammad AO , Arora S , Rodrigues S , et al. Malnutrition and sarcopenia predict post‐liver transplantation outcomes independently of the Model for End‐stage Liver Disease score. J Cachexia Sarcopenia Muscle 2017;8:113–121. 27239424 10.1002/jcsm.12095 PMC4864202

[54] Solheim TS , Laird BJA , Balstad TR , Stene GB , Bye A , Johns N , et al. A randomized phase II feasibility trial of a multimodal intervention for the management of cachexia in lung and pancreatic cancer. J Cachexia Sarcopenia Muscle 2017;8:778–788. 28614627 10.1002/jcsm.12201 PMC5659068

[55] Boengler K , Kosiol M , Mayr M , Schulz R , Rohrbach S . Mitochondria and ageing: role in heart, skeletal muscle and adipose tissue. J Cachexia Sarcopenia Muscle 2017;8:349–369. 28432755 10.1002/jcsm.12178 PMC5476857

[56] van Dijk DPJ , Bakens MJAM , Coolsen MME , Rensen SS , van Dam RM , Bours MJL , et al. Low skeletal muscle radiation attenuation and visceral adiposity are associated with overall survival and surgical site infections in patients with pancreatic cancer. J Cachexia Sarcopenia Muscle 2017;8:317–326. 27897432 10.1002/jcsm.12155 PMC5377384

[57] Nijholt W , Scafoglieri A , Jager‐Wittenaar H , Hobbelen JSM , van der Schans CP . The reliability and validity of ultrasound to quantify muscles in older adults: a systematic review. J Cachexia Sarcopenia Muscle 2017;8:702–712. 28703496 10.1002/jcsm.12210 PMC5659048

[58] van Vugt JLA , Levolger S , Gharbharan A , Koek M , Niessen WJ , Burger JWA , et al. A comparative study of software programmes for cross‐sectional skeletal muscle and adipose tissue measurements on abdominal computed tomography scans of rectal cancer patients. J Cachexia Sarcopenia Muscle 2017;8:285–297. 27897414 10.1002/jcsm.12158 PMC5697014

[59] Rutten IJG , Ubachs J , Kruitwagen RFPM , Beets‐Tan RGH , Olde Damink SWM , Van Gorp T . Psoas muscle area is not representative of total skeletal muscle area in the assessment of sarcopenia in ovarian cancer. J Cachexia Sarcopenia Muscle 2017;8:630–638. 28513088 10.1002/jcsm.12180 PMC5566632

[60] Martone AM , Bianchi L , Abete P , Bellelli G , Bo M , Cherubini A , et al. The incidence of sarcopenia among hospitalized older patients: results from the Glisten study. J Cachexia Sarcopenia Muscle 2017;8:907–914. 28913934 10.1002/jcsm.12224 PMC5700449

[61] Holeček M . Beta‐hydroxy‐beta‐methylbutyrate supplementation and skeletal muscle in healthy and muscle‐wasting conditions. J Cachexia Sarcopenia Muscle 2017;8:529–541. 28493406 10.1002/jcsm.12208 PMC5566641

[62] Snijders T , Nederveen JP , Joanisse S , Leenders M , Verdijk LB , van Loon LJC , et al. Muscle fibre capillarization is a critical factor in muscle fibre hypertrophy during resistance exercise training in older men. J Cachexia Sarcopenia Muscle 2017;8:267–276. 27897408 10.1002/jcsm.12137 PMC5377411

[63] Nishikawa H , Enomoto H , Ishii A , Iwata Y , Miyamoto Y , Ishii N , et al. Elevated serum myostatin level is associated with worse survival in patients with liver cirrhosis. J Cachexia Sarcopenia Muscle 2017;8:915–925. 28627027 10.1002/jcsm.12212 PMC5700437

[64] Brown JL , Rosa‐Caldwell ME , Lee DE , Blackwell TA , Brown LA , Perry RA , et al. Mitochondrial degeneration precedes the development of muscle atrophy in progression of cancer cachexia in tumour‐bearing mice. J Cachexia Sarcopenia Muscle 2017;8:926–938. 28845591 10.1002/jcsm.12232 PMC5700433

[65] Morley JE . Anorexia of ageing: a key component in the pathogenesis of both sarcopenia and cachexia. J Cachexia Sarcopenia Muscle 2017;8:523–526. 28452130 10.1002/jcsm.12192 PMC5566640

[66] Bye A , Sjøblom B , Wentzel‐Larsen T , Grønberg BH , Baracos VE , Hjermstad MJ , et al. Muscle mass and association to quality of life in non‐small cell lung cancer patients. J Cachexia Sarcopenia Muscle 2017;8:759–767. 28493418 10.1002/jcsm.12206 PMC5659054

[67] St‐Jean‐Pelletier F , Pion CH , Leduc‐Gaudet J‐P , Sgarioto N , Zovilé I , Barbat‐Artigas S , et al. The impact of ageing, physical activity, and pre‐frailty on skeletal muscle phenotype, mitochondrial content, and intramyocellular lipids in men. J Cachexia Sarcopenia Muscle 2017;8:213–228. 27897402 10.1002/jcsm.12139 PMC5377417

[68] Baracos VE . Psoas as a sentinel muscle for sarcopenia: a flawed premise. J Cachexia Sarcopenia Muscle 2017;8:527–528. 28675689 10.1002/jcsm.12221 PMC5566635

[69] Gonzalez MC , Heymsfield SB . Bioelectrical impedance analysis for diagnosing sarcopenia and cachexia: what are we really estimating? J Cachexia Sarcopenia Muscle 2017;8:187–189. 28145079 10.1002/jcsm.12159 PMC5377383

[70] Mochamat Cuhls H , Marinova M , Kaasa S , Stieber C , Conrad R , Radbruch L , et al. A systematic review on the role of vitamins, minerals, proteins, and other supplements for the treatment of cachexia in cancer: a European Palliative Care Research Centre cachexia project. J Cachexia Sarcopenia Muscle 2017;8:25–39. 27897391 10.1002/jcsm.12127 PMC5326814

[71] Tournadre A , Pereira B , Dutheil F , Giraud C , Courteix D , Sapin V , et al. Changes in body composition and metabolic profile during interleukin 6 inhibition in rheumatoid arthritis. J Cachexia Sarcopenia Muscle 2017;8:639–646. 28316139 10.1002/jcsm.12189 PMC5566648

[72] dos Santos L , Cyrino ES , Antunes M , Santos DA , Sardinha LB , Sarcopenia and physical independence in older adults: the independent and synergic role of muscle mass and muscle function . J Cachexia Sarcopenia Muscle 2017;8:245–250. 27897417 10.1002/jcsm.12160 PMC5377449

[73] von Haehling S , Morley JE , Coats AJS , Anker SD . Ethical guidelines for publishing in the *Journal of Cachexia, Sarcopenia and Muscle*: update 2019. J Cachexia Sarcopenia Muscle 2019;10:1143–1145. 31661195 10.1002/jcsm.12501 PMC6818444

